# Management of macular edema with branch retinal vein occlusion in a case of secondary polycythemia 

**DOI:** 10.3205/oc000127

**Published:** 2019-11-20

**Authors:** Sumeer Singh, Srividya Neriyanuri, Rajiv Raman

**Affiliations:** 1 Elite School of Optometry, Chennai, India; 2 Shri Bhagawan Mahavir Vitreoretinal Services, Sankara Nethralaya Hospital, Chennai, India

**Keywords:** polycythemia, macular edema, branch retinal vein occlusion, intravitreal injections

## Abstract

**Purpose:** We report a case of polycythemia with an ocular complication of branch retinal vein occlusion associated with macular edema that was managed by anti-vascular endothelial growth factor (VEGF) and systemic management.

**Methods:** A 43-year-old, one-eyed male, a known case of polycythemia presented with complaints of decreased vision in the right eye. He underwent comprehensive eye evaluation, fundus photography and optical coherence tomography at the baseline visit and post intravitreal ranibizumab 1-, 3-, 4- and 11-month follow-up.

**Results:** A patient with polycythaemia was diagnosed in the right eye with superotemporal branch retinal vein occlusion and macular edema, which clinically and on optical coherence tomography resolved after 1 intravitreal injection of ranibizumab. However, as he discontinued systemic management, macular edema reappeared and the edema resolved well after intravitreal ranibizumab. He then became more compliant to the systemic therapy and was asymptomatic for the last 7 months.

**Conclusion:** In a case of retinal vein occlusion with macular edema of recognizable cause, the management of systemic disease and anti-VEGF can give satisfactory results.

## Introduction

Polycythemia vera or primary polycythemia is a myeloproliferative disorder characterized by the presence of increased red blood cells, haemoglobin and total blood volume and occurs due to a JAK2 mutation [1]. Haematological alterations in primary polycythemia give rise to prothrombosis, an event that results in serious ocular and systemic manifestations [2], [3]. Secondary polycythemia is a heterogeneous group of disorders which results in abnormal increase in blood cell mass due to a decrease in tissue oxygenation and alterations in production of erythropoietin. Previous reports have shown a presence of bilateral retinal vein occlusion as initial manifestation of polycythemia [4]. There has been paucity of information on ocular complications in patients with secondary polycythemia. 

We report a case of secondary polycythemia who presented to us with unilateral branched retinal vein occlusion with macular edema and was managed by phlebotomy and intravitreal ranibizumab. The case highlights the importance of treatment of the systemic disease in reducing the need of intravitreal injections and implications in recurrence of macular edema. To the best of our knowledge, macular edema with branch retinal vein occlusion (BRVO) as a complication of secondary polycythemia was not reported earlier.

## Case description

A 43-year-old male presented to a tertiary eye care centre with complaints of sudden onset (3 days) blurring of vision in the right eye. He was one-eyed and had lost his left eye 20 years ago following a traumatic injury. He was a heavy smoker and gave a positive history of alcohol consumption. He was a known case of polycythemia since 6 months; he was negative for JAK2 mutation and had high haematocrit values (Table 1 (Tab. 1)) indicating a secondary polycythemia according to the guidelines set by the British Committee for Standards in Haematology [5]. He was on systemic anti-hypertensive treatment and was advised phlebotomy. However, he was non-compliant to phlebotomy.

On clinical examination, his best corrected visual acuity (BCVA) was 6/7.5 for distance and N6 for near in the right eye. Anterior segment evaluation was normal and intraocular pressure by applanation tonometry was 14 mm Hg in the right eye. Dilated fundus evaluation revealed a superotemporal branch retinal vein occlusion (ST BRVO) with macular edema. Cirrus high definition optical coherence tomography (Carl Zeiss Meditec, Germany) using macular cube (A scans 512, B scans 128) and 5-line raster scans (4096 A-scans) showed an increased central subfield foveal thickness (358 microns) with the presence of cystoid macular edema (CME) and subretinal fluid (SRF). As the patient was symptomatic, even though his vision was good, he was advised intravitreal anti-VEGF and a review with his physician for phlebotomy. The patient underwent intravitreal injection of ranibizumab (0.5 mg) and was advised to undergo phlebotomy regularly.

**Figure 1:**
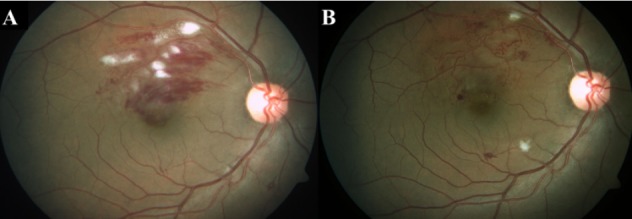
Fundus pictures A: Fundus picture of the right eye showing a superotemporal branch retinal vein occlusion at the baseline visit. B: Fundus picture at 1^st^ follow-up, a month after anti-VEGF showing resolved superotemporal branch retinal vein occlusion.

On th consecutive 1^st^ and 2^nd^ monthly follow-up visits, the patient was asymptomatic, his BCVA was 6/6 for distance and N6 for near. Optical coherence tomography (OCT) showed a normal central subfield foveal thickness (180 microns) with resolved CME and SRF (Figure 1 (Fig. 1)). 

At the 3^rd^ monthly visit, the patient complained of a sudden drop in vision. He was noncompliant to the treatment advised by his physician for secondary polycythemia. On ocular examination, there was a drop in BCVA to 6/18 with recurrence of CME (increased central subfield foveal thickness – 521 microns). He underwent intravitreal injection of ranibizumab (0.5 mg) at this visit. He was explained the need for compliance to systemic management. On the subsequent visit, the patient’s vision improved to 6/6, N6 and OCT showed resolving macular edema. 

**Figure 2:**
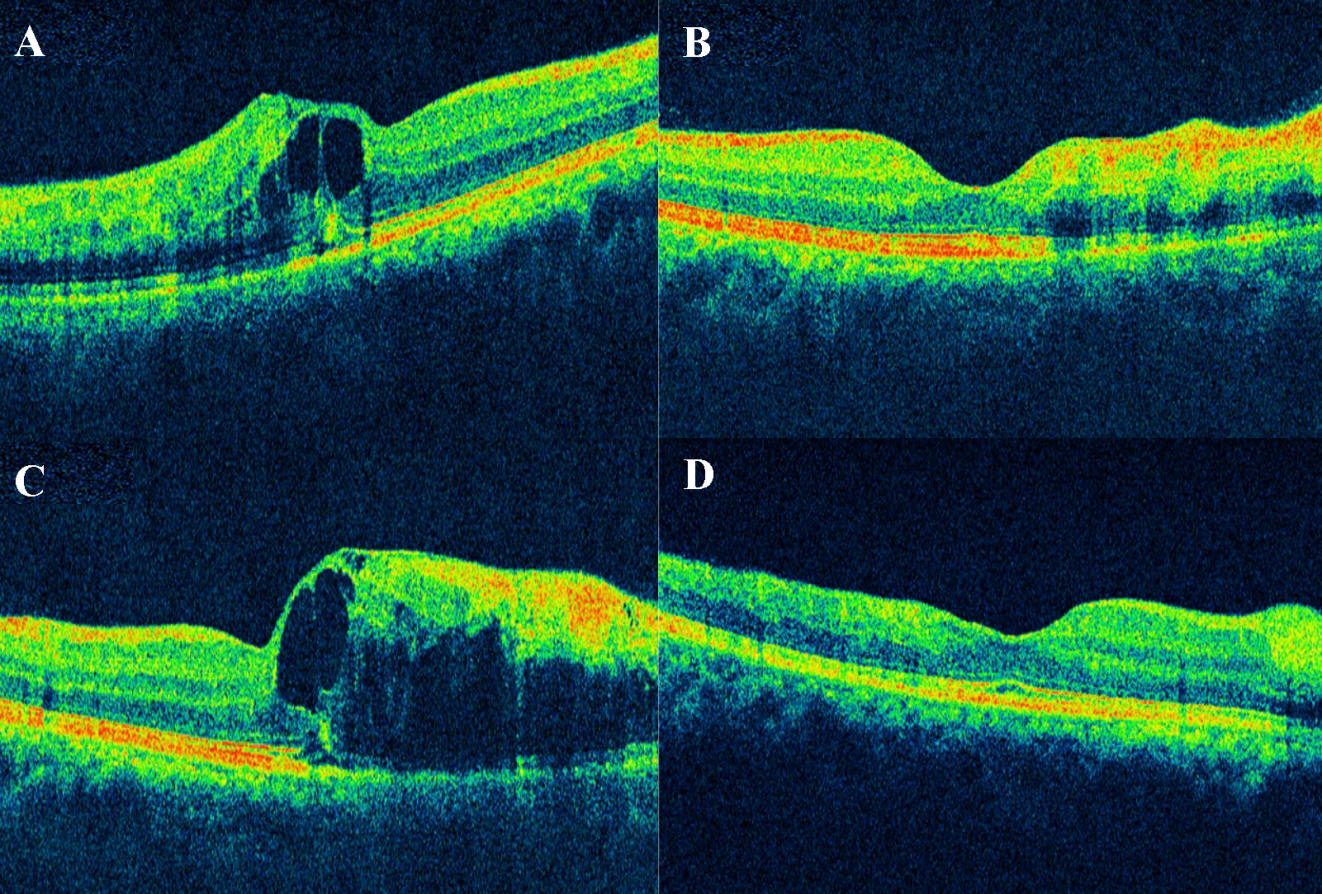
Optical coherence tomography (OCT) A: OCT at the baseline visit shows an increased foveal contour with macular edema, photoreceptor layer and retinal pigment epithelium (RPE) layer are disrupted temporal to the fovea. B: OCT at the 1^st^ follow-up, a month after anti-VEGF shows resolved macular edema. C: OCT at the 3^rd^ monthly follow-up shows reoccurrence of the macular edema with sub retinal fluid noted. D: OCT at the last visit, 11-month follow-up from the baseline shows completely resolved macular edema, normal inner and outer retinal layers noted.

Thereafter, he was followed up monthly for 3 months and bimonthly for 1 year. On, the 1-year follow-up, the patient maintained a BCVA of 6/6, N6 with no recurrence of vein occlusion or macular edema (Figure 2 (Fig. 2)). He was under regular care of his treating physician for secondary polycythemia.

## Discussion

Ocular complications of polycythemia include retinal and choroidal ischemia, retinal haemorrhages, papilloedema and cilioretinal artery occlusion, all of which pose a risk of visual loss and transient blindness [2], [6]. Phlebotomy, a bloodletting procedure for polycythemia, also reduces the risk of systemic and ocular morbidity [6], [7].

Systemic management together with an appropriate ocular management for macular edema and vascular occlusions plays a pivotal role in eye salvage. Previous studies demonstrate that intravitreal ranibizumab therapy is effective for the reduction of macular edema and improvement in visual acuity. PRN (pro re nata) dosing, treat and extend regimen are also effective in treating macular edema in BRVO rather than having monthly injections [8] [9]. In the present case, timely management of BRVO and strict adherence to the systemic management helped in the recovery of lost vision and halted the reoccurrence of macular edema. This case report brings out an important observation that in cases where the cause of BRVO is certain, treatment of the underlying cause is crucial as it helps to reduce the number of ocular injections a patient receives.

Thus, BRVO with macular edema secondary to hematological diseases like polycythemia can be managed by systemic control of the disease and as needed injection of intravitreal anti-VEGF agents. However, as there are issues of safety due to the hypercoagulable state of the blood, they need regular physician care and monitoring.

## Notes

### Competing interests

The authors declare that they have no competing interests.

## Figures and Tables

**Table 1:**
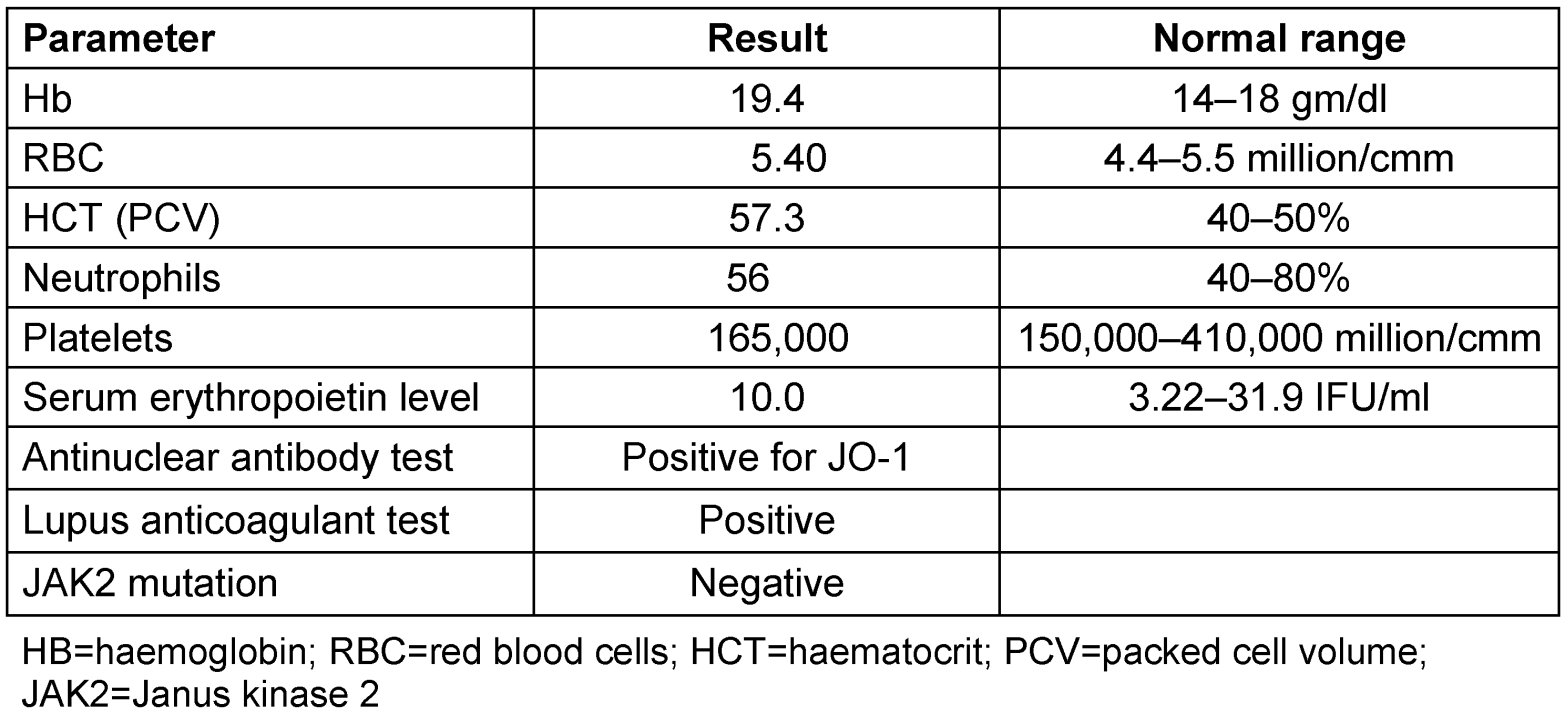
Baseline laboratory evaluation for polycythemia
